# Calcium-Sensing Receptor Participates in High Glucose-Induced EndMT in Primary Human Aortic Endothelial Cells

**DOI:** 10.3389/fphys.2020.629542

**Published:** 2021-01-15

**Authors:** Cheng Yuan, Lihua Ni, Xianqin Yang, Changjiang Zhang, Xiaoyan Wu

**Affiliations:** ^1^Department of Gynecological Oncology, Zhongnan Hospital of Wuhan University, Wuhan, China; ^2^Department of Nephrology, Zhongnan Hospital of Wuhan University, Wuhan, China; ^3^Department of Emergency, Central Hospital of Enshi Tujia and Miao Autonomous Prefecture, Enshi, China; ^4^Department of Cardiology, Renmin Hospital of Wuhan University, Wuhan, China

**Keywords:** calcium-sensing receptor, high glucose, endothelial to mesenchymal transition, human aortic endothelial cell, bioinformatics analysis

## Abstract

**Objective:**

Previous studies have shown that high glucose (HG) induces endothelial cell (EC) damage via endothelial-to-mesenchymal transition (EndMT). Although the underlying mechanisms are still unclear, recent studies have demonstrated the role of calcium-sensing receptor (CaSR) in mediating EC damage. Therefore, the aim of our study was to investigate whether CaSR mediates HG-induced EndMT and to determine the underlying mechanism.

**Methods:**

Bioinformatics analysis of microarray profiles (GSE30780) and protein-protein interaction (PPI) analyses were performed to select the hub genes. As for *in vitro* research, the human aortic ECs (HAECs) were exposed to HG to induce EndMT. The expression of CaSR and β-catenin was determined, as well as their effects on EndMT (endothelial marker CD31, mesenchymal marker FSP1, and α-SMA).

**Results:**

The bioinformatics analysis indicated CaSR was significantly increased in HG-treated HAECs and was one of the hub genes. The *in vitro* results showed that HG significantly inhibited the expression of CD31 and increased FSP1 and α-SMA in a concentration- and time-dependent manner. Moreover, CaSR was increased in HAECs after HG treatment. The CaSR antagonist attenuated HG-induced expression of EndMT-related markers. Furthermore, HG treatment increased the nuclear translocation of β-catenin in HAECs. In contrast, blocking the nuclear translocation of β-catenin by DKK1 could attenuate HG-induced EndMT (increased the protein expression of CD31 by 30% and decreased the protein expression of FSP1 by 15% and α-SMA by 25%). CaSR siRNA further inhibited the HG-induced nuclear translocation of β-catenin in HAECs.

**Conclusion:**

Our research demonstrated that HG-induced EndMT in HAECs might be mediated by CaSR and the downstream nuclear translocation of β-catenin.

## Introduction

Cardiovascular deteriorations are some of the most serious complications in diabetes mellitus (DM) (Strain and Paldánius, 2018; Provenzano et al., 2019; Schlieper, 2020). High levels of serum glucose is believed to be the key to promoting the association between endothelial damage and cardiovascular disease (Giugliano et al., 2019; Joffre et al., 2020; Liberale et al., 2020). Previous studies have shown that high glucose (HG) induces endothelial cell (EC) damage via endothelial-to-mesenchymal transition (EndMT) (Maleszewska et al., 2013; Liguori et al., 2019), which contributes to endothelial injury and subsequent cardiovascular disease in DM. However, the underlying mechanisms of HG-induced EndMT remain largely unclear.

Calcium-sensing receptor (CaSR) is a member of the G-protein-coupled receptor family, which is involved in the synthesis and secretion of parathyroid hormone (Chavez-Abiega et al., 2020). In addition, CaSR is expressed in ECs, cardiomyocytes, and vascular smooth muscle cells (Hannan et al., 2018). Recent evidences suggested CaSR activation may be related to further EC damage, and blocking CaSR may prevent EC damage (Xiao et al., 2017; Xie et al., 2018). These findings suggested the participation of CaSR in cardiovascular diseases. Interestingly, it remains unclear whether CaSR is involved in HG-induced EndMT.

β-catenin is a central component of the adherens junctions and transcriptional effector of Wnt signaling, acting through pools with different subcellular localizations (membrane vs. cytoplasmic/nuclear pool, respectively) (McCrea et al., 2015). The canonical Wnt/β-catenin pathway is a potent inducer of EndMT, as indicated by the translocation of β-catenin to the nucleus, where it acts as a transcription factor. Our previous studies showed that elevated parathyroid hormone levels induced EndMT in cultured human aortic ECs, which was associated with the canonical Wnt/β-catenin pathway (Wu et al., 2017; Ni et al., 2019). In addition, a study established directional crosstalk between CaSR and Wnt/β-catenin signaling in keratinocyte differentiation (Popp et al., 2014). Whether the nuclear translocation of β-catenin is a downstream effector of the HG/CaSR pathway under HG stimulation in ECs remains unclear.

Overall, we hypothesized that HG can induce EndMT via a CaSR-dependent pathway, which accounts for cardiovascular disease in DM. In this study, we examined the effects of HG on EndMT in human aortic endothelial cells (HAECs) and investigated the underlying mechanism of CaSR regulation.

## Materials and Methods

### Transcriptome Microarray Data Analysis

The raw genes expression data of GSE30780 was downloaded from the National Center of Biotechnology Information (NCBI) GEO^1^ database. This dataset comprised between the HAEC samples exposed to HG and the matched NG samples, and was generated from samples using the GPL570 Affymetrix human genome GPL6884 (Illumina HumanWG-6 v3.0) expression beadchip. The original data from the series matrix files and the corresponding probe annotation information were downloaded and converted into a detectable format. The background correction and normalization of the data were conducted by the Robust Multi-Array Average method, and the missing values were inserted by the k-Nearest Neighbor method. After preprocessing, the limma package was used to screen the differentially expressed genes (DEGs) between the HAECs samples exposed to HG and the matched NG samples with a *t*-test. The adjusted *P*-value accounted for false discovery rates (FDR) and was calculated by the Benjamini and Hochberg method. Hierarchical clustering was used to qualitatively analyze all of the DEGs from the microarray data and place them into two groups using the heatmap package. Search Tool for the Retrieval of Interacting Genes (STRING)^2^ online database, a biological predictive web resource including numerous proteins and known interactive functions, was employed to analyze and evaluate the interaction correlations among DEGs. Cytoscape software was then used to construct the protein-protein interaction (PPI) network according to the information from STRING.

### Cell Culture and Reagents

The HAECs were purchased from ScienCell Research Laboratories (No. 6100) and cultured as previously described (Xie et al., 2018). Passage 2–6 HAECs were selected for our study. At approximately 75% confluence, we changed the culture medium to a serum-free solution for 12 h prior to further experiments. To examine the effects of HG on EndMT in HAECs, cells were treated with HG (Sigma) at different concentrations [normal glucose (NG) 5.5, HG 15, and HG 30 mmol/l] and times (0, 24, and 48 h). Calhex (Sigma, 10 μM) and dickkopf (DKK1) (PeproTech, 500 ng/ml) were then added to the serum-free medium to study the effect of loss of function of CaSR and β-catenin on EndMT.

### Western Blot

Equal amounts of protein were obtained from cell lysates, electrophoresed on SDS-polyacrylamide gel and transferred onto a nitrocellulose membrane (Pall) by electroblotting. Blots were incubated overnight with primary antibodies against CD31 (sc-376764, Santa Cruz, 1:1,000 dilution), FSP1 (ab-197896, Abcam, 1:1,000 dilution), CaSR (ab-19347, Abcam, 1:1,000 dilution), and GAPDH (ab-181602, Abcam, 1:1,000 dilution) followed by secondary antibodies (7074s, CST, 1:2,000 dilution or 7076s, CST, 1:2,000 dilution). In addition, the nuclear proteins were prepared by a nuclear and cytoplasmic protein extraction kit (KeyGen). Blots were incubated overnight with primary antibody against β-catenin (ab-32572, Abcam, 1:2,000 dilution). Lamin B served as the internal control for nuclear proteins.

### Quantitative Real-Time PCR (qRT-PCR)

Total RNA was extracted using RNAiso Plus based on the manufacturer’s protocols (TAKARA). Next, the concentration and purity were detected by a Nanodrop 2000 (Thermo). GAPDH was used as a reference for normalization. The relative mRNA values were calculated by the 2^–ΔΔCt^ method. The primers are presented in Table 1.

**TABLE 1:** Primer sequences used for real-time PCR analysis.

**Gene**	**Sequence or target sequence (5′–3′)**
CD31-F	TGGGGTTGTCTTTGAATACCG
CD31-R	TCATAAAAGGACAGACCATCG
FSP1-F	GTCCACCTTCCACAAGTAC
FSP1-R	TGTCCAAGTTGCTCATCAG
CaSR-F	AGGA AAGGGATCATTGAGGG
CaSR-R	ATGCAGGAGGTGTGGTTCTC
CaSR-siRNA	CGGGGTACCTTAAGCACCTACGGCATCTAA (sense)
	GCTCTAGAGTTAACGCGATCCCAAAGGGCTC (antisense)
GAPDH-F	GGAGCGAGATCCCTCCAAAAT
GAPDH-R	GGCTGTTGTCATACTTCTCATGG

### Immunofluorescence Staining

Coverslips were fixed with 4% paraformaldehyde for 15 min. The cells were then immunostained with primary antibody against CaSR (ab-19347, Abcam, 1:250 dilution) at 4°C overnight. Then, the cells were incubated with a fluorescent secondary antibody (A-31570, Invitrogen, 1:300 dilution) for 1 h in the dark and finally counterstained with DAPI (G1012, Servicebio).

### Small Interfering RNA (siRNA) Transfection

siRNA targeting CaSR was obtained from GenePharma. The transfection reagent Lipo 2000 was acquired from Invitrogen (No: 12566014). The cells were transfected based on the transfection reagent manufacturer’s instructions as previously described (Wen et al., 2018).

### Statistical Analysis

Bioinformatics adopted R 3.5.1, and cut-off value of the DEGs was |log_2_ FC| ≥ 1 and adjusted *P* < 0.05. Data of vitro experiment were expressed as the means ± *SD* and analyzed by one-way analysis of variance (ANOVA) using SPSS 20.0 software. The results were considered significant if *P* < 0.05. Experiments were repeated at least three times.

## Results

### Bioinformatic Analysis

**FIGURE 1:**
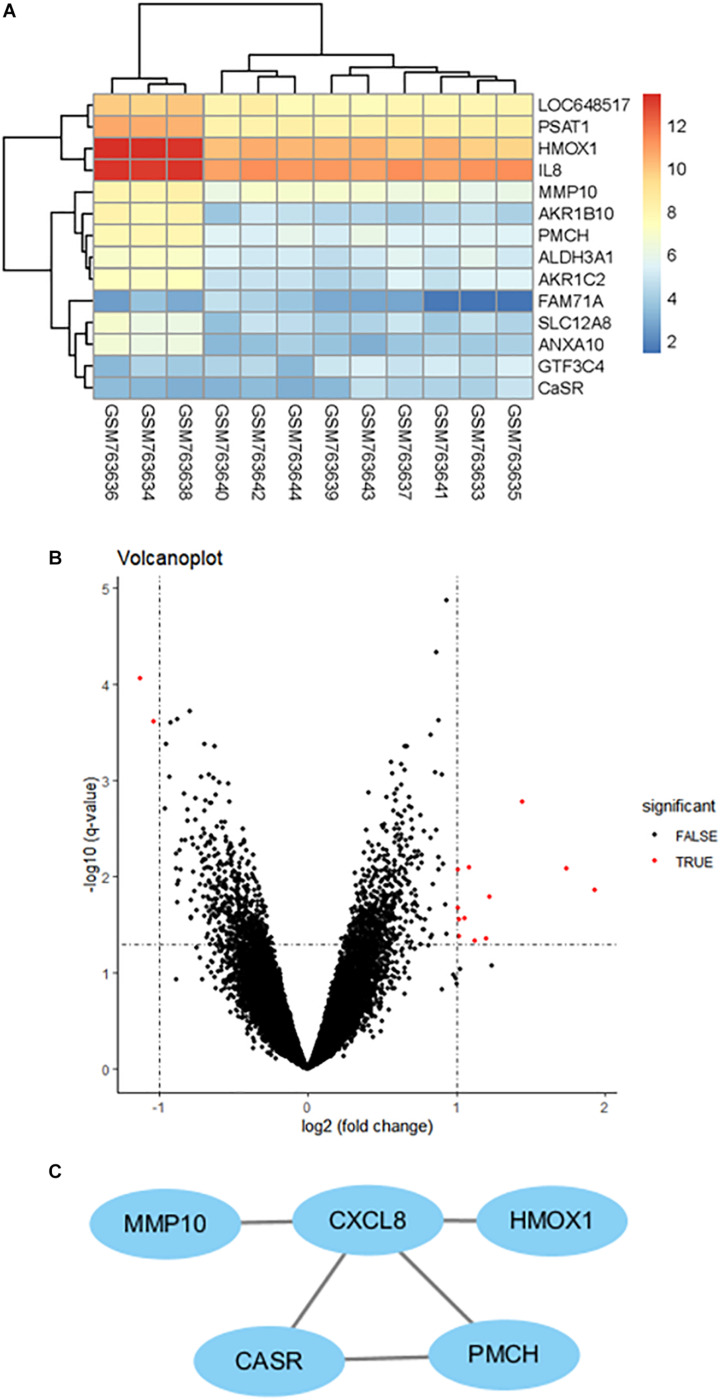
Clustering of the differentially expressed genes (DEGs) between the HAEC samples exposed to HG and the matched NG samples. **(A)** Heatmap of DEGs between the HG group and the NG group. Each column represents a sample, and each row represents the expression level of an mRNA. **(B)** Volcano plot of DEGs. **(C)** Protein–protein interaction network of differentially expressed genes (DEGs) based on the STRING online database.

A total of 12 HAEC samples were divided into 6 HG and the matched 6 NG samples. After integrated analysis in the microarray data, a total of 14 DEGs were screened (Figure 1A). Volcano plots (Figure 1B) were generated to show the correlation between DEGs. To identify the interactions and key genes of the DEGs, a PPI network was constructed according to information from the STRING online database (Figure 1C). The results indicated CaSR was significantly increased in HG-treated HAECs and was one of the hub genes.

### HG Induced EndMT in Cultured HAECs

**FIGURE 2:**
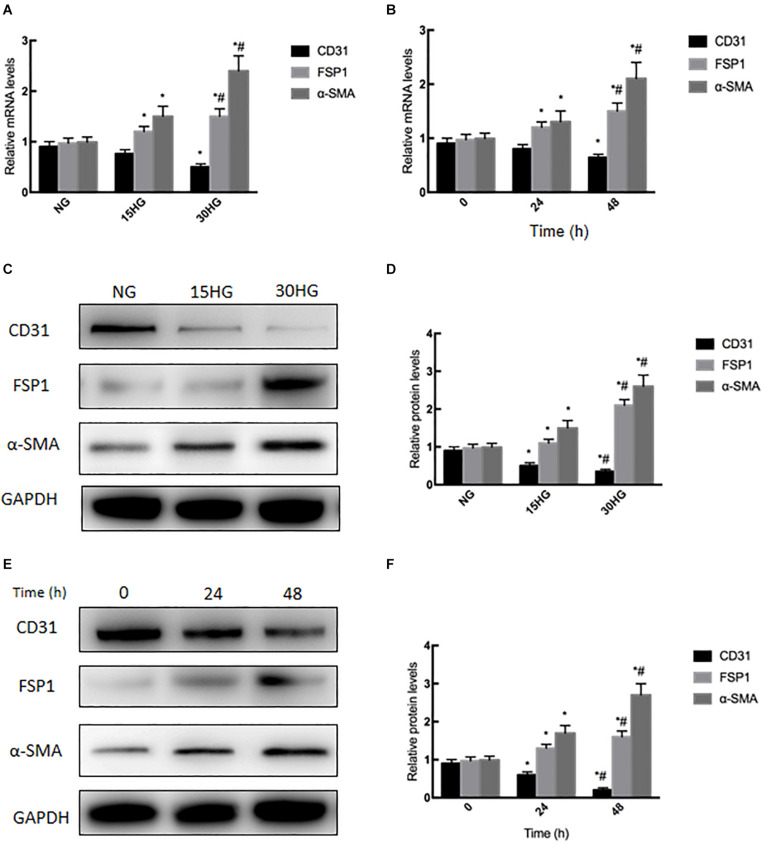
Exposure to HG induces EndMT in HAECs. HAECs were cultured with increasing concentrations of glucose (5.5, 15, 30 mmol/l) for 48 h. **(A,C,D)** mRNA and protein levels of CD31, FSP1 and α-SMA were detected by qRT-PCR and western blot; next, we cultured HAECs with HG (30 mmol/l) for different time periods (0, 24, 48 h). **(B,E,F)** mRNA and protein levels of CD31, FSP1, and α-SMA were detected via qRT-PCR and western blot; data are presented as the means ± *SD* and analyzed by one-way analysis of variance (ANOVA). *P < 0.05 vs. the NG group or 0 h group. ^#^*P* < 0.05 vs. the 15HG group or 24 h group. Experiments were repeated three times. NG, normal glucose (5.5 mmol/l). HG, high glucose (30 mmol/l).

To further study the effects of HG on EndMT, we treated HAECs with elevated levels of glucose (NG: 5.5, HG: 15, and HG: 30 mmol/l). With increasing concentrations, HG significantly decreased the mRNA expression of CD31 (endothelial marker) and increased FSP1 (mesenchymal marker), which is characteristic of EndMT (Figure 2A). In addition, the cells were incubated with HG (30 mmol/l) for different times (0, 24, and 48 h). We observed that with prolonged treatment time, HG significantly decreased the mRNA expression of CD31, while increasing FSP1 and α-SMA expression (Figure 2B). As determined by western blotting, HG reduced the protein expression of CD31 and increased the expression of FSP1 and α-SMA in a concentration- and time-dependent manner (Figures 2C–F). The above results revealed that HG induced EndMT in cultured HAECs.

### HG Upregulated CaSR Expression in HAECs

**FIGURE 3:**
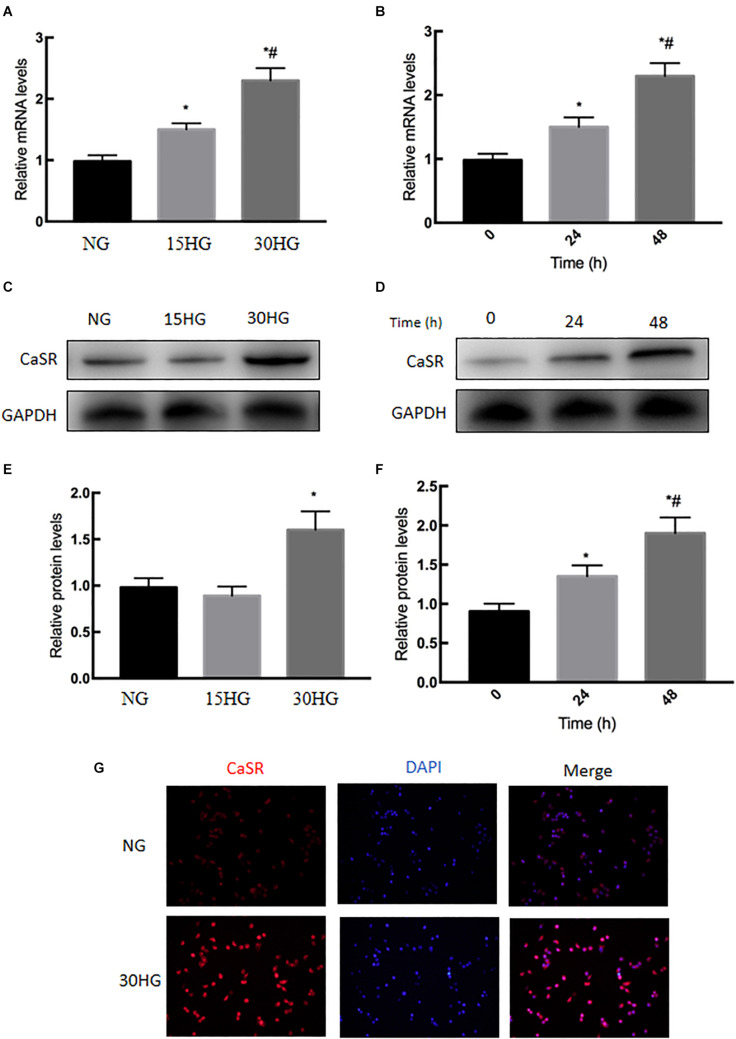
Effects of HG on CaSR mRNA and protein expression in HAECs. HAECs were cultured with increasing concentrations of glucose (5.5, 15, 30 mmol/l) for 48 h. **(A)** mRNA and **(C,E)** protein levels of CaSR were detected by qRT-PCR and Western blot; next, we further incubated HAECs with HG (30 mmol/l) for different time periods (0, 24, 48 h). **(B)** mRNA and **(D,F)** protein levels of CaSR were detected by qRT-PCR and Western blot. **(G)** Representative immunofluorescence images demonstrating CaSR (red) staining in HAECs treated with NG or HG (30 mmol/l) for 48 h. The nuclei were stained with DAPI (blue). Data are presented as the means ± *SD* and analyzed by one-way analysis of variance (ANOVA). *P < 0.05 vs. the NG group or 0 h group. ^#^*P* < 0.05 vs. the 15HG group or 24 h group. Experiments were repeated three times. NG, normal glucose (5.5 mmol/l). HG, high glucose (30 mmol/l).

We next examined the expression level of CaSR under HG stimulation in cultured HAECs. As shown in Figures 3A–F, qRT-PCR and western blot analyses indicated that the mRNA and protein levels of CaSR were upregulated in a dose- and time-dependent manner with HG treatment. In addition, immunofluorescence staining revealed that CaSR expression was increased after HG treatment (Figure 3G). These phenomena suggested the activation of CaSR in HG-treated HAECs.

### Blocking CaSR Inhibited HG-Induced EndMT in HAECs

**FIGURE 4:**
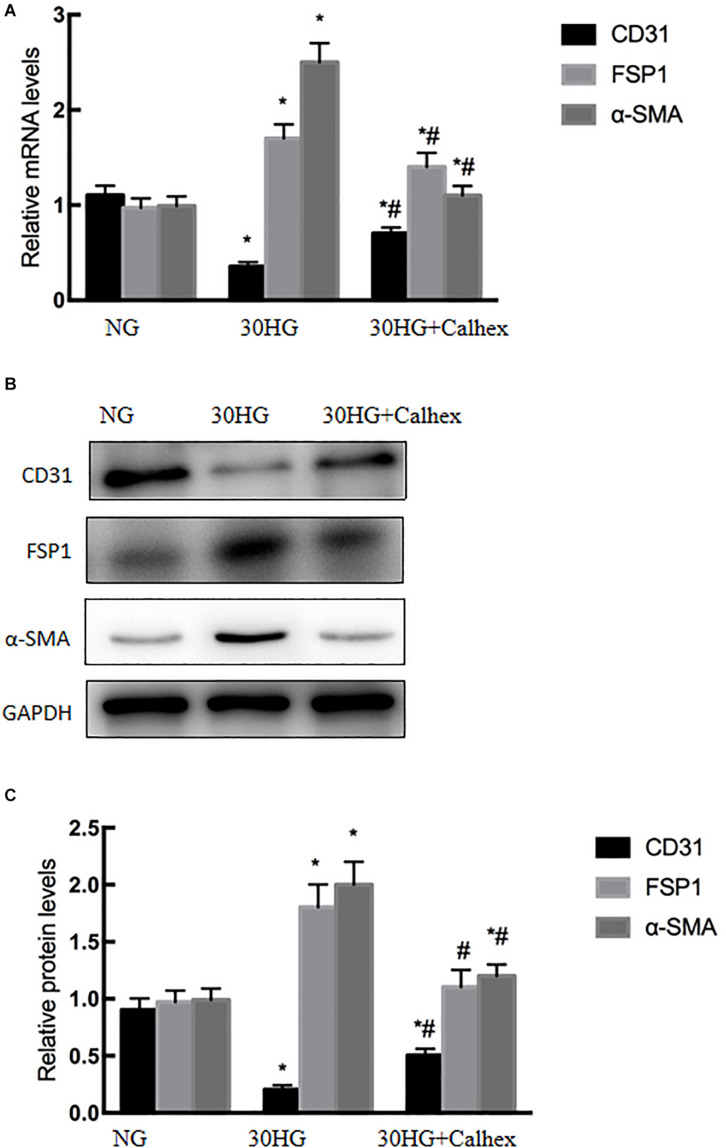
Blocking CaSR inhibited HG-induced EndMT in HAECs. Calhex, a classical CaSR antagonist, was used in the study. HAECs were incubated with Calhex (10 μmol/l, 48 h) in the presence of HG. **(A)** The mRNA levels of CD31, FSP1, and α-SMA were measured by qRT-PCR. **(B)** Representative blots for CD31, FSP1, and α-SMA. **(C)** Graphic presentation. Data are presented as the means ± *SD* and analyzed by one-way analysis of variance (ANOVA). *P < 0.05 vs. the NG group,^ #^*P* < 0.05 vs. the HG group. Experiments were repeated three times. NG, normal glucose (5.5 mmol/l). HG, high glucose (30 mmol/l).

To confirm the effects of CaSR on HG-induced EndMT, HAECs were incubated with HG (30 mmol/l, 48 h) in the presence or absence of the CaSR antagonist Calhex231. As shown in Figures 4A–C, qRT-PCR and western blot analysis showed that blocking CaSR with Calhex effectively alleviated the HG-induced decrease in CD31 expression and the HG-induced increase in FSP1 and α-SMA expression. These data suggested that HG-induced EndMT might be mediated by CaSR.

### HG Activated the Nuclear Translocation of β-Catenin in HAECs

**FIGURE 5:**
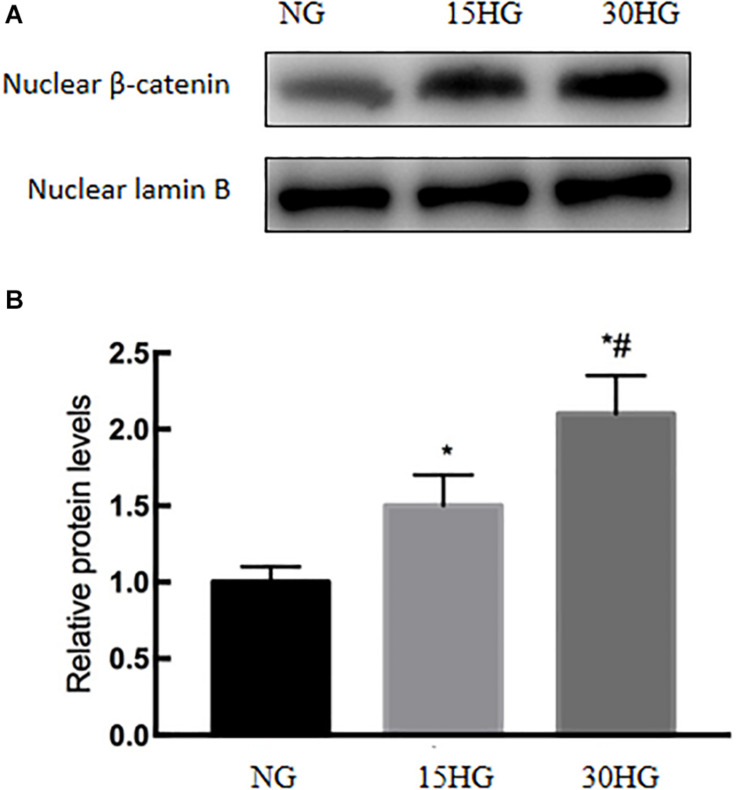
HG activated the nuclear translocation of β-catenin in HAECs. HAECs were cultured with increasing concentrations of glucose (5.5, 15, 30 mmol/l) for 48 h. **(A)** Representative blots showing the nuclear expression of β-catenin. **(B)** Graphic presentation. Data are presented as the means ± *SD* and analyzed by one-way analysis of variance (ANOVA). *P < 0.05 vs. the NG group,^ #^*P* < 0.05 vs. the 15HG group. Experiments were repeated three times. NG, normal glucose (5.5 mmol/l). HG, high glucose (30 mmol/l).

Next, we further investigated the mechanisms involved in HG-induced EndMT, and we detected the nuclear expression of β-catenin in HAECs. As the concentration of glucose increased, the nuclear protein level of β-catenin also increased (Figure 5), which indicated the nuclear translocation of β-catenin.

### Blocking the Nuclear Translocation of β-Catenin Inhibited HG-Induced EndMT in HAECs

**FIGURE 6:**
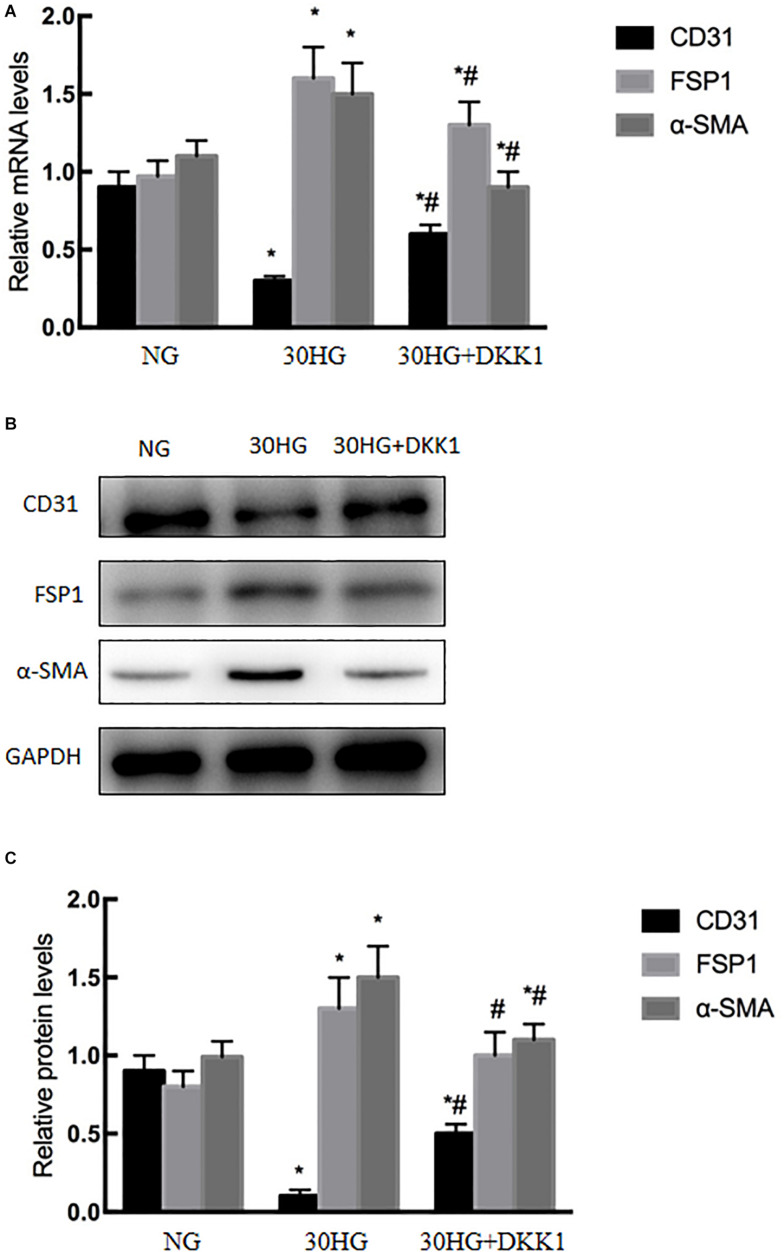
Blocking the nuclear translocation of β-catenin inhibited HG-induced EndMT in HAECs. DKK1, a classical β-catenin antagonist, was used in the study. HAECs were incubated with DKK1 (500 g/ml, 48 h) in the presence of HG. **(A)** The mRNA levels of CD31, FSP1, and α-SMA were measured by qRT-PCR. **(B)** Representative blots for CD31, FSP1 and α-SMA. **(C)** Graphic presentation. Data are presented as the means ± *SD* and analyzed by one-way analysis of variance (ANOVA). *P < 0.05 vs. the NG group,^ #^*P* < 0.05 vs. the HG group. Experiments were repeated three times. NG, normal glucose (5.5 mmol/l). HG, high glucose (30 mmol/l).

DKK1, a classic antagonist of β-catenin activation, was applied to HAECs. As shown in Figure 6, the expression level of CD31 was significantly upregulated in the HG + DKK1 group compared with the HG group, while the expression of FSP1 and α-SMA was downregulated. These data revealed that the nuclear translocation of β-catenin was involved in HG-induced EndMT in HAECs.

### CaSR Blockade Suppressed the HG-Induced Nuclear Translocation of β-Catenin in HAECs

**FIGURE 7:**
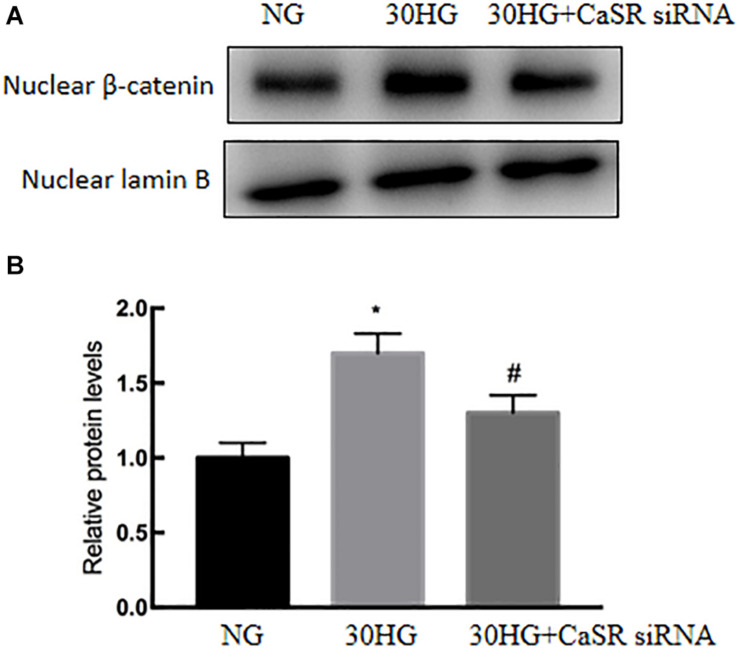
CaSR blockade suppressed the HG-induced nuclear translocation of β-catenin in HAECs. siRNA, specific to CaSR, was used. **(A)** Protein levels of nuclear β-catenin were detected by Western blot. **(B)** Results of quantitative protein levels. The results are presented as the means ± *SD* and analyzed by one-way analysis of variance (ANOVA). *P < 0.05 vs. the NG group,^ #^*P* < 0.05 vs. the HG group. Experiments were repeated three times. NG, normal glucose (5.5 mmol/l). HG, high glucose (30 mmol/l).

Next, we further investigated the roles of CaSR in HG-induced nuclear translocation of β-catenin in HAECs. As shown in Figure 7, the nuclear expression level of β-catenin was significantly upregulated in the HG group, while CaSR siRNA transfection markedly suppressed nuclear β-catenin relocation.

## Discussion

Several researchers have proven the activation of CaSR in the development and progression of cardiovascular diseases (Liu et al., 2019; Yuan et al., 2019; Zhang et al., 2019b). However, it remains unclear whether it involved in DM. To our knowledge, our present study is the first time to combine bioinformatics and *in vitro* study to investigate HG induced EndMT and to determine the underlying mechanism. In the end, we found that CaSR was significantly increased in HG treated HAECs and one of the hub genes based on the PPI analysis. We think this work will attract the attention of a broad readership due to the range of vascular disease in DM.

DM is recognized to increase the risk of cardiovascular diseases (Kovacic et al., 2014; Henning, 2018; Luo et al., 2019). Numerous complex molecular mechanisms have been proposed to investigate the structural and functional alternations in the cardiovascular deterioration, including EC damage (Fiorentino et al., 2013; Petrie et al., 2018; Scheen, 2018). As we known, EC damage is considered to be the early site, accelerates atherosclerosis and subsequently causes cardiovascular events. Emerging evidence has suggested that HG induced EndMT is involved in the onset and progression of EC damage (Geng and Guan, 2017; Shang et al., 2017; Zhang et al., 2019a). Guan et al. demonstrated that miR-448-3p alleviates diabetic vascular dysfunction by inhibiting EndMT through DPP-4 dysregulation (Guan et al., 2020). Tang et al. proved that Angiotensin II mediates the HG induced EndMT *in vitro*, which was inhibited by Irbesartan (Tang et al., 2010). Our previous study revealed that Ephrin B2 mediates HG induced EndMT in human aortic endothelial cells through FAK pathway (Yuan et al., 2020). The results of our recently study demonstrated that HG-induced EndMT was accompanied by increased endothelial CaSR expression (Figures 2, 3), which was firstly further supported by the bioinformatics analysis (Figure 1). In addition, blockade of CaSR by Calhex was found to significantly inhibit EndMT (Figure 4). These results are in agreement with studies showing increased expression of CaSR in calcified tissues (Issa et al., 2019). Taken together, these findings showed a crucial role for CaSR activation in EC injury in DM.

CaSR activation triggers different intracellular pathways. Fang et al. (2020) showed that the ERK signaling pathway is involved in CaSR activation in human intrahepatic cholangiocarcinoma. Zhao et al. (2020) proved that CaSR induced the proliferation of osteosarcoma cells by activating the PI3K-Akt pathway. Zhang et al. (2019b) found that CaSR participated in the regulation of vascular tension in the mesentery of hypertensive rats via the PLC-IP3/AC-V/cAMP/RAS pathway. Interestingly, many studies proved that β-catenin was required for EndMT, while EndMT was inhibited after TGF-β2 stimulation in β-catenin-deficient ECs (Liebner et al., 2004; Zhong et al., 2018; Sabbineni et al., 2019). In our current study, we further evaluated whether the nuclear translocation of β-catenin is involved in CaSR-mediated EndMT in HG-treated HAECs. As the data show, we observed that elevated HG increased the translocation of β-catenin (Figure 5), and CaSR siRNA could partially attenuate this change in HAECs (Figure 7). Then, we found that blockade of nuclear β-catenin expression could further inhibit EndMT induced by HG (Figure 6). Overall, these results proved that nuclear translocation of β-catenin might be a downstream effector of the HG/CaSR pathway under HG stimulation.

Our study was the first to combine bioinformatics techniques and *in vitro* study to investigate HG-induced EndMT and to determine the underlying mechanism. Then we proved that CaSR regulated HG-induced EndMT in HAECs. Interestingly, several researches had also studied the mechanism of HG-induced EndMT. Angiotensin II, Ephrin B2 were involved in HG-induced EndMT in HAECs (Tang et al., 2010; Yuan et al., 2020). And the mechanism of HG-induced EndMT were complicated. CaSR might participate in HG-induced EndMT and inhibition of CaSR only partly abolished the HG-induced EndMT. Besides, our study found that CaSR mediated HG-induced EndMT by regulating β-catenin signaling, which was supported by several previous studies. Rybchyn et al. (2011) reported that activation of the CaSR in human osteoblasts resulted in the AKT-dependent translocation of β-catenin to the nucleus. Wu et al. (2018) proved that activation of the CaSR attenuated the IL-1β-induced translocation of β-catenin. Bhagavathula et al. (2007) demonstrated that regulation of β-catenin in colon carcinoma is dependent on CaSR expression and function. Thus, CaSR could regulate the β-catenin phosphorylation, which lead to the HG-induced EndMT.

However, our study has some limitations. First, the dose of the inhibitor [Calhex (CaSR inhibitor) and DKK1] in our study was a low dose that could achieve the research goal. The concentrations of the inhibitors were selected based on many related articles (Awumey et al., 2013; Wu et al., 2014, 2017; Xie et al., 2014; Jang et al., 2017). We performed concentration-response curves for the inhibitors, which are included in Supplementary Figure 1. We found that 10 μM Calhex could achieve 60% knockout and 500 ng/ml DKK1 achieved 65% knockout. In general, these inhibitory effects were sufficient to perform the loss-of-function study and are widely accepted for basic research. Second, siRNA technology cannot achieve 100% knockout effects at present (Yamamura et al., 2019). The siRNA treatment is a routine method for loss-of-function studies. In our study, siRNA technology downregulated the expression level of the target gene by more than 60%. In general, such knockdown effects are widely acceptable in basic research. Third, the study is limited to HAECs. As we know, HAECs are not ideal models for adult endothelial cells. However, HAECs are widely used cell lines for research on inflammation-related endothelial dysfunction and vascular calcification (Wu et al., 2016, 2017; Chen et al., 2018; Kostina et al., 2019). HAECs are commonly recognized as a substitute for the basic research study of endothelial cells. Fourth, numerous researches had proved that HG-induced EndMT contributed to endothelial damage (Maleszewska et al., 2013; Liguori et al., 2019; Guan et al., 2020), which lead to cardiovascular diseases in diabetes. Thus, our study focus on the role of CaSR in HG-induced EndMT, which might underlie the mechanism cardiovascular diseases in diabetes.

**FIGURE 8:**
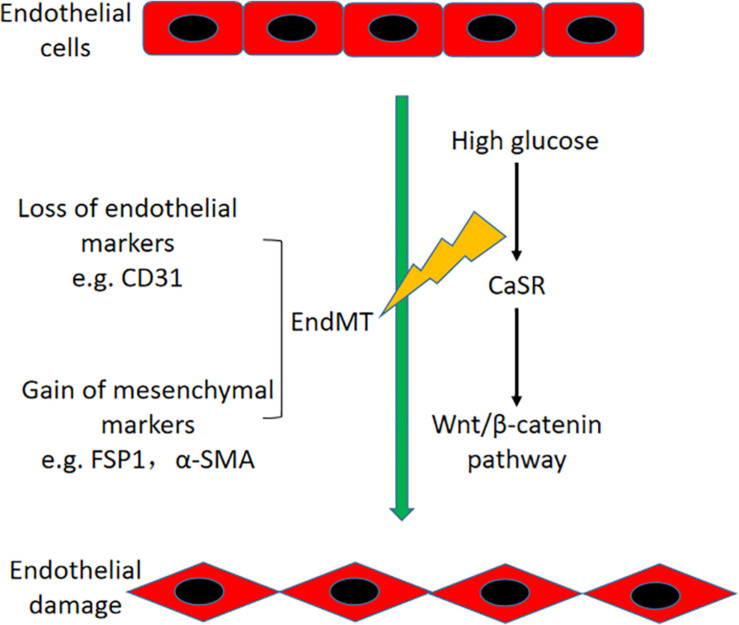
Schematic diagram of this study. HG-induced EndMT is regulated by CaSR and the downstream nuclear translocation of β-catenin in HAECs.

In conclusion, our study showed that HG-induced EndMT is partially mediated by CaSR and the downstream nuclear translocation of the β-catenin pathway in HAECs (Figure 8). These findings may provide an improved understanding of the CaSR-dependent pathway, which might lead to a novel therapeutic target for vascular disease in DM.

## Data Availability Statement

The raw data supporting the conclusions of this article will be made available by the authors, without undue reservation, to any qualified researcher.

## Author Contributions

CY, LHN, and XQY performed the research. XYW designed the research study. LHN and CJZ contributed essential reagents or tools. CJZ analyzed the data. CY, LHN, and XQY wrote the manuscript. All the authors contributed to the article and approved the submitted version.

## Conflict of Interest

The authors declare that the research was conducted in the absence of any commercial or financial relationships that could be construed as a potential conflict of interest.

## Footnotes

1 http://www.ncbi.nlm.nih.gov/geo/

2 https://string-db.org/

## Supplementary Material

Supplementary Figure 1 Concentration-response-curves for CaSR and β-catenin. HAECs were cultured with increased concentration of Calhex (0, 2.5, 5, 10 μM) or DKK1 (0, 62, 125, 250, 500 ng/ml) for 48 h. The mRNA levels of CaSR and β-catenin were measured by qRT-PCR. Data were exhibited as means ± *SD*. ^∗^*P* < 0.05 vs. 0 group, ^∗∗^*P* < 0.01 vs. 0 group. Experiments were repeated three times.

Supplementary Figure 2 Effects of Calhex 231 in EndMT. HAECs were cultured in the presence and absence of Calhex (10 μM) for 48 h. The mRNA levels of EndMT-related markers were measured by qRT-PCR. Data were exhibited as means ± *SD* and analyzed by one-way analysis of variance (ANOVA). Experiments were repeated three times.
