# Supplementary figures and images for: Calcium-Sensing Receptor Participates in High Glucose-Induced EndMT in Primary Human Aortic Endothelial Cells
Source: Front Physiol. 2021 Jan 15;11:629542. doi: 10.3389/fphys.2020.629542 (PMC7844313; doi:10.3389/fphys.2020.629542)

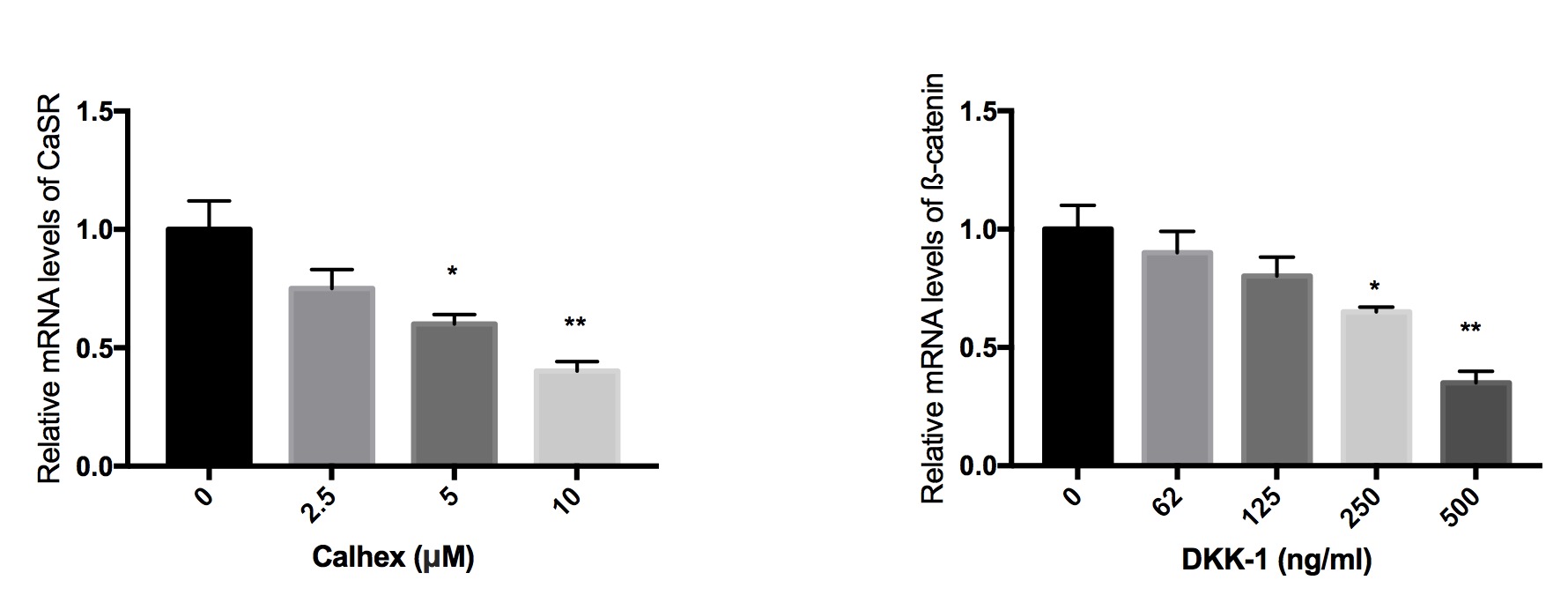

Supplement: Supplementary Figure 1 — Concentration-response-curves for CaSR and β-catenin. HAECs were cultured with increased concentration of Calhex (0, 2.5, 5, 10 μM) or DKK1 (0, 62, 125, 250, 500 ng/ml) for 48 h. The mRNA levels of CaSR and β-catenin were measured by qRT-PCR. Data were exhibited as means ± SD. ∗P < 0.05 vs. 0 group, ∗∗P < 0.01 vs. 0 group. Experiments were repeated three times. [file Image_1.JPEG]

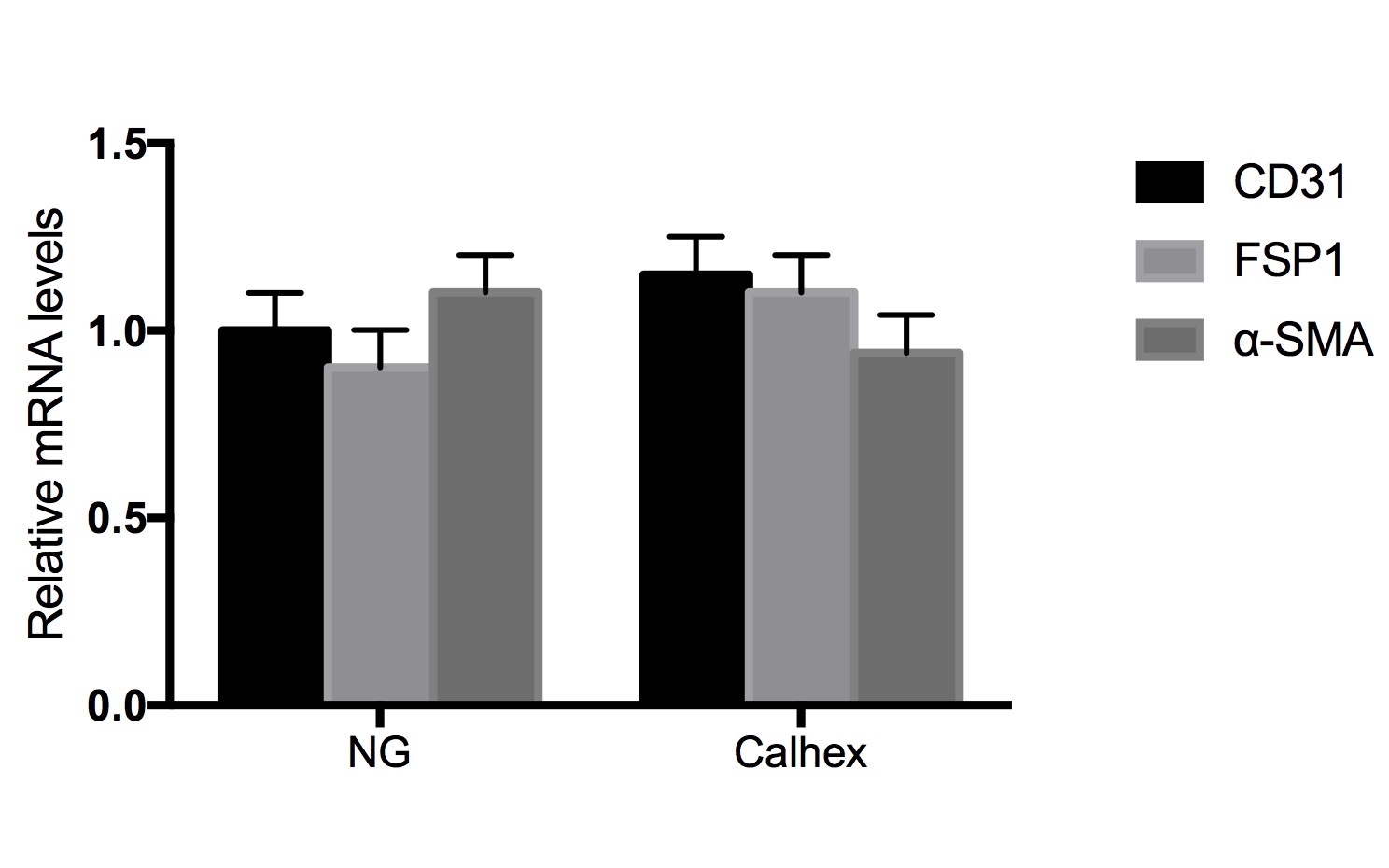

Supplement: Supplementary Figure 2 — Effects of Calhex 231 in EndMT. HAECs were cultured in the presence and absence of Calhex (10 μM) for 48 h. The mRNA levels of EndMT-related markers were measured by qRT-PCR. Data were exhibited as means ± SD and analyzed by one-way analysis of variance (ANOVA). Experiments were repeated three times. [file image_2.jpeg]
